# Effectiveness of a structured life skills training program among Indian nursing students

**DOI:** 10.6026/973206300200740

**Published:** 2024-07-31

**Authors:** Buvaneswari Ramakrishnan, Juliet Sylvia

**Affiliations:** 1 Department of Psychiatric Nursing, KMCH College of Nursing, Coimbatore, Tamilnadu - 641048, India; 2 Department of Community health Nursing, Alagappa College of Nursing, Karaikudi, Tamilnadu - 630003, India

**Keywords:** Nursing education, life skills training, first-year students, intervention, and effectiveness

## Abstract

Nursing education programs increasingly emphasize the importance of life skills alongside clinical competencies to prepare students for the multifaceted demands of modern healthcare. However, there remains a gap in understanding the most effective strategies for integrating life skills into nursing curricula. Therefore, it is of interest to evaluate the impact of a structured life skills training program on first-year B.Sc. Nursing students' proficiency in selected life skills in Tamil Nadu, India. A true experimental pretest-posttest design was employed, with 257 first-year B.Sc. Nursing students from six selected colleges in Trichy district, Tamil Nadu, participating. The intervention, an eight-week Life Skills Training Program, was delivered to the experimental group, while the control group received no intervention. Data were collected using a self-administered questionnaire comprising socio-demographic variables and the Life Skills Assessment Scale. Statistical analysis included chi-square tests, one-way ANOVA repeated measures, and correlation analysis. Significant differences were observed between the experimental and control groups in various life skills, including self-awareness, empathy, interpersonal relationships, and creative thinking (p < 0.001). Age, place of stay, number of friends, hobbies, reason for choosing nursing, and interest in the course showed significant associations with pretest life skills scores (p < 0.05). Positive correlations were found between certain life skills, emphasizing their interconnectedness (p < 0.05). The structured life skills training program demonstrated effectiveness in enhancing the life skills proficiency of first-year B.Sc. Nursing students. The study underscores the importance of integrating life skills development into nursing education curricula to better prepare students for the complexities of patient care. Tailored interventions based on individual characteristics may further optimize outcomes. Future research should explore longitudinal effects and additional factors influencing life skills development among nursing students.

## Background:

In the dynamic landscape of healthcare, the role of nurses extends beyond clinical expertise to encompass a holistic approach that integrates psychosocial well-being with physical care. Life Skills have been central to Health Promotion interventions and programmes with children and adolescents for over 40 years. [1] Recognizing this, contemporary nursing education programs emphasize the development of life skills alongside clinical competencies to equip nursing students with the resilience, adaptability, and interpersonal skills necessary for effective patient care. [2] Despite the acknowledged importance of life skills, there remains a gap in understanding the optimal strategies for their cultivation within nursing education. [3] The undergraduate nursing program is demanding, especially for mental health nursing students who encounter significant stressors such as working with individuals in distress. The Decider Life Skills, a one-day training program, aims to enhance resilience and coping skills to support students in managing professional and personal challenges during practice placements. [4] The transition from student nurse to practicing professional requires a multifaceted skill set encompassing not only clinical knowledge but also effective communication, critical thinking, and problem-solving abilities. These life skills are essential for navigating the complexities of patient care, fostering therapeutic relationships, and promoting patient-centered care practices. In an era marked by rapid technological advancements, globalization, and evolving healthcare paradigms, the need for adaptable and resourceful nurses has become increasingly evident. [5] Study on effectiveness of such interventions in enhancing the life skills proficiency of nursing students remains an area of ongoing inquiry. Therefore, it is of interest to evaluate the impact of a structured life skills training program on selected life skills among first-year B.Sc. Nursing students in selected nursing colleges in Tamil Nadu.

## Methodology:

## Research design:

The present study adopts a true experimental pretest-posttest.[6]

## Research setting:

The study is conducted in nursing colleges in Tamil Nadu, specifically in Trichy district. Six nursing colleges were randomly selected from Trichy, ensuring representation from various areas to prevent contamination among the sample.

## Sample and sample Size:

The target population comprises first year B.Sc. Nursing students in selected nursing colleges in Trichy district. The accessible population includes those students who meet the inclusion criteria. The sample size estimation utilized a statistical formula for comparing two independent means, resulting in a minimum sample size of 108 in each group. Ultimately, the final sample size consists of 126 subjects in the experimental group and 131 subjects in the control group.

## Sampling technique:

Multi-stage cluster sampling is employed for the study.[7] Trichy district is selected first, followed by the random selection of nursing colleges within the district. Six colleges are chosen through simple random sampling. Each nursing college represents one cluster.

## Intervention:

The intervention, the Life Skills Training Program, was conducted over a period of eight weeks. Each session was two hours long, and a total of eight sessions were held throughout the duration of the program. The training sessions were implemented consistently on the same day of each week to maintain continuity and ensure maximum participation and engagement from the first-year B.Sc. Nursing students. This structured approach allowed for comprehensive coverage of various life skills topics and provided ample time for participants to absorb the knowledge and practice the skills taught during the sessions.

## Data collection tool:

The primary data collection tool used is a self-administered questionnaire consisting of two parts:

[1] Socio-demographic variables of first year B.Sc. Nursing students.

[2] Life Skills Assessment Scale [LSAS], a standardized tool developed and validated for assessing life skills.

## Ethical Considerations:

Several ethical considerations are addressed:

[1] Approval from the dissertation committee and ethical clearance from the selected nursing college are obtained.

[2] Administrative permission is secured from authorities of the nursing colleges.

[3] Informed consent is obtained from each participant, ensuring understanding and voluntary participation.

[4] Anonymity and confidentiality of data are assured.

## Results:

Table 1 succinctly encapsulates the demographic and attitudinal characteristics of sampled first-year B.Sc. Nursing students. The majority were aged 17 or 18, with fewer aged 19. A notable proportion in the experimental group resided in hostels, contrasting with the control group. Moreover, a significant majority of experimental group students reported having more than five friends, while playing games was more common among the control group. Additionally, more students in the experimental group cited personal interest as their reason for choosing nursing and showed a higher interest in the course compared to the control group. Table 2 examines the association between pre-test life skills scores and socio-demographic variables of first-year B.Sc. Nursing students. Significant associations are found for certain variables. For instance, age shows no significant association with self-awareness, empathy, effective communication, or interpersonal relationship scores. However, empathy scores exhibit a significant association with age (Kw Test, p=0.029), indicating that older students tend to demonstrate higher empathy levels. Moreover, hobbies show significant associations with various life skills, such as self-awareness (Kw Test, p=0.024) and effective communication (Kw Test, p=0.045), indicating that students with certain hobbies tend to score higher in these areas. Table 3 presents the results of a one-way ANOVA repeated measures test for global life skills scores of the experimental and control groups. The data indicate significant differences between the two groups across all measured life skills except for effective communication. Specifically, the experimental group demonstrates significantly higher scores than the control group in self-awareness (Experimental: F=46.09, p<0.001; Control: F=7.35, p<0.001), empathy (Experimental: F=47.129, p<0.001; Control: F=10.400, p<0.001), interpersonal relationship (Experimental: F=52.706, p<0.001; Control: F=15.478, p<0.001), and creative thinking (Experimental: F=132.41, p<0.001; Control: F=65.78, p<0.001). These findings highlight the effectiveness of the intervention in enhancing various life skills among nursing students, emphasizing its potential impact on their overall development and readiness for professional practice. Regarding relationships between mean pretest life skills of first year B.Sc. Nursing students in the experimental group the correlations between mean pretest life skills among first-year B.Sc. Nursing students in the experimental group. Significant positive correlations are observed between self-awareness and empathy [r=0.266, p<0.01], as well as between self-awareness and effective communication [r=0.206, p<0.05]. Additionally, a significant positive correlation is found between empathy and effective communication [r=0.194, p<0.05]. These findings emphasize the interconnectedness of different life skills and highlight their relevance in nursing education.

## Discussion:

In our study, we observed significant improvements in various life skills among first-year B.Sc. Nursing students following the implementation of a structured life skills training program. This finding is consistent with the results reported in the study by Yang *et al.* [2018], where a similar intervention led to positive outcomes among nursing students.[8] Our study findings align with those of the study by avatar Iran *et al.* [2019], which reported significant enhancements in empathy and effective communication skills among nursing students following a life skills training program [9]. Similarly, the study by Zeydani *et al.* [2020], through a meta-analysis of multiple studies, concluded that life skills training programs have a positive impact on various domains of nursing students' skill development. [10] These consistent results across different studies reinforce the effectiveness of life skills training programs are promoting our study, we discovered significant associations between socio-demographic variables and pretest life skills scores among first-year B.Sc. Nursing students. Notably, older students displayed higher levels of empathy, while certain hobbies were linked to stronger life skills such as self-awareness and effective communication. These findings suggest that individual characteristics may influence the development of life skills in nursing education. Comparing our results with previous studies, our findings align with research indicating the impact of age and personal interests on life skills development. For instance, a comparative study by Suaad *et al.*, found similar associations between age and empathy levels among nursing students, supporting our findings. [11] Additionally, research by Batool *et al.* [2019] highlighted the importance of hobbies in shaping students' communication skills, reinforcing our observation of hobbies' influence on effective communication abilities. [12]

## Conclusion:

The significant associations between socio-demographic variables and life skills proficiency highlight the need for tailored interventions in nursing education. Moving forward, future research could explore longitudinal effects and additional factors influencing life skills development among nursing students.

## Figures and Tables

**Table 1:** Distribution & comparison of the socio-demographic variables of first year B.Sc. Nursing students in the experimental and control group N=257

**Variables**	**Control[131]**		**Experimental[126]**		**Chi- square value**	**p-value**
	**No.**	**%**	**No.**	**%**		
1. Age in years						
17	61	46.6	68	54		
18	61	46.6	52	41.3	1.6	0.449
19	9	6.9	6	4.8		
2. Place of stay						
Hostel	101	77.1	123	97.6		
Home	30	22.9	3	2.4	24.163	<0.001***
3. Number of friends						
1-2	16	12.2	6	4.8		
3-5	25	19.1	8	6.3	15.608	<0.001***
>5	90	68.7	112	88.9		
4. Hobbies						
Reading books	34	26	34	27		
Listening to music	15	11.5	21	16.7		
Dancing	12	9.2	13	10.3	5.917	0.205
Drawing	30	22.9	13	10.3		
Playing games	40	30.5	23	18.3		
5. Medium of instruction in higher secondary education						
Tamil	108	82.4	99	78.6	0.614	0.433
English	23	17.6	27	21.4		
6. Reason for choosing nursing						
Own interest	96	73.3	108	85.7		
Parents force	25	19.1	11	8.7	6.585	0.037*
Other reasons	10	7.6	7	5.6		
7. Interest in the course						
Interested	121	92.4	119	94.4	0.449	0.503
Not interested	10	7.6	7	5.6		
*Statistically significant, *** p<0.001, * p<0.05

**Table 2:** Association between Pretest Life Skills Scores and Socio-demographic Variables of First Year B.Sc. Nursing Students

**Socio-demographic Variable**	**Self-awareness [Kw Test, p-value]**	**Empathy [Kw Test, p-value]**	**Effective Communication [Kw Test, p-value]**	**Interpersonal Relationship [Kw Test, p-value]**
Age	0.005 [0.942]	4.762* [0.029]	0.115 [0.735]	0.766 [0.381]
Place of stay	0.256 [0.880]	2.850 [0.241]	0.654 [0.721]	1.829 [0.401]
Number of Friends	4.171 [0.124]	3.280 [0.194]	3.819 [0.148]	0.118 [0.943]
Hobbies	11.284* [0.024]	4.491 [0.344]	25.625*** [0.000]	6.084 [0.193]
Medium of Instruction	0.212 [0.645]	6.341* [0.012]	0.104 [0.747]	2.477 [0.116]
Reason for Choosing Nursing	5.765 [0.056]	1.395 [0.498]	4.636 [0.098]	0.188 [0.910]
Interest in the Course	6.896* [0.009]	3.676 [0.055]	4.023* [0.045]	4.519*[0.034]
*Significant at p<0.05 level

**Table 3:** One way ANOVA repeated measures test for Global Life Skills Scores of the experimental and control group N=257

**Measure**	**Group**	**F-value**	**p-value**
Self-awareness	Experimental	46.09	<0.001***
	Control	7.35	<0.001***
Empathy	Experimental	47.129	<0.001***
	Control	10.4	<0.001***
Effective Communication	Experimental	25.841	<0.001***
	Control	1.266	0.284
Interpersonal Relationship	Experimental	52.706	<0.001***
	Control	15.478	<0.001***
Creative Thinking	Experimental	132.41	<0.001***
	Control	65.78	<0.001***
